# Design and Evaluation of the Initial 50th Percentile Female Prototype Rear Impact Dummy, BioRID P50F – Indications for the Need of an Additional Dummy Size

**DOI:** 10.3389/fbioe.2021.687058

**Published:** 2021-07-16

**Authors:** Anna Carlsson, Johan Davidsson, Astrid Linder, Mats Y. Svensson

**Affiliations:** ^1^Chalmers Industrial Technology (Chalmers Industriteknik), Gothenburg, Sweden; ^2^Vehicle Safety Division, Chalmers University of Technology, Gothenburg, Sweden; ^3^Swedish National Road and Transport Research Institute (VTI), Gothenburg, Sweden

**Keywords:** crash test dummy, females, rear impact, sled testing, soft tissue neck injury, vehicle safety, volunteer tests, whiplash

## Abstract

The objective of this study was to present the design of a prototype rear impact crash test dummy, representing a 50th percentile female, and compare its performance to volunteer response data. The intention was to develop a first crude prototype as a first step toward a future biofidelic 50th percentile female rear impact dummy. The current rear impact crash test dummy, BioRID II, represents a 50th percentile male, which may limit the assessment and development of whiplash protection systems with regard to female occupants. Introduction of this new dummy size will facilitate evaluation of seat and head restraint (HR) responses in both the average sized female and male in rear impacts. A 50th percentile female rear impact prototype dummy, the BioRID P50F, was developed from modified body segments originating from the BioRID II. The mass and rough dimensions of the BioRID P50F is representative of a 50th percentile female. The prototype dummy was evaluated against low severity rear impact sled tests comprising six female volunteers closely resembling a 50th percentile female with regard to stature and mass. The head/neck response of the BioRID P50F prototype resembled the female volunteer response corridors. The stiffness of the thoracic and lumbar spinal joints remained the same as the average sized male BioRID II, and therefore likely stiffer than joints of an average female. Consequently, the peak rearward angular displacement of the head and T1, and the rearward displacement of the T1, were lesser for the BioRID P50F in comparison to the female volunteers. The biofidelity of the BioRID P50F prototype thus has some limitations. Based on a seat response comparison between the BioRID P50F and the BioRID II, it can be concluded that the male BioRID II is an insufficient representation of the average female in the assessment of the dynamic seat response and effectiveness of whiplash protection systems.

## Introduction

Vehicle crashes causing Whiplash Associated Disorder (WAD) are still of worldwide concern. Despite new seat designs intended to lessen the risk of whiplash injury and Advanced Driver Assistance Systems (ADAS) that reduces the number of rear impacts, the long-term consequences of whiplash injuries remain (Kullgren et al., 2020). Cars equipped with advanced whiplash protection systems posed on average a ∼50% lower risk of long-term whiplash injuries in comparison to cars equipped with standard seats (Davidsson and Kullgren, 2013). According to a review by Carlsson, 2012, accident data have shown that females typically have twice the risk of sustaining whiplash injuries than males, even under similar crash conditions.

In rear impacts, the whiplash injury risk in cars equipped with conventional seats generally shows a growing trend for increasing statures for both females and males, where tall females are associated with the greatest risk (Temming and Zobel, 1998; Jakobsson et al., 2000). It is however important to note that the greatest whiplash injury frequencies are associated with females and males of average statures (Carlsson et al., 2014). Based on Swiss and Swedish insurance records, Carlsson et al. (2014) concluded that the stature and mass of the females most frequently injured, correspond reasonably well with the average stature and mass of females in the European countries.

Today, rear impact testing is performed with 50th percentile male dummies, mainly the BioRID II, which may limit the assessment and development of whiplash protection systems since the female part of the population is not represented. In terms of stature and mass, the 50th percentile male crash test dummy roughly corresponds to the 90th–95th percentile female (Welsh and Lenard, 2001), resulting in females not being adequately represented by the BioRID II. Previous studies show that the BioRID II matches 50th percentile male volunteer responses (Davidsson et al., 1999, 2000). However, more recent volunteer studies show that the response of 50th percentile females is clearly different to 50th percentile males (Linder et al., 2008; Carlsson et al., 2010, 2011; Carlsson, 2012). Similar differences were found in a comparison between the BioRID II and a prototype rear impact crash test dummy, representing a 50th percentile female, Schmitt et al. (2012). The BioRID II is thus not adequately representative of 50th percentile females. Since the male BioRID II has only been validated with regard to tests with male volunteers, current seats are assessed without consideration of female properties, despite a higher whiplash injury risk in females. This limitation may contribute to whiplash protection systems being more effective for males than for females. According to insurance claims records (Kullgren and Krafft, 2010), the risk reduction for permanent medical impairment was approximately 30% greater for males than for females. In recent years, injury statistics show that whiplash injuries still present a major problem, and that the whiplash injury risk females are exposed to is substantially higher (Kullgren et al., 2020).

The objective of this study was to present the design of the prototype rear impact crash test dummy, representing a 50th percentile female, used in Schmitt et al. (2012). Furthermore, the performance of this prototype dummy was compared to volunteer response data. The intention was to develop a first crude prototype as a first step toward a future biofidelic 50th percentile female rear impact dummy. Introduction of this new dummy size will facilitate evaluation of seat and head restraint (HR) responses in both the average sized female and male in rear impacts.

## Materials and Methods

A rear impact dummy prototype, called BioRID P50F, representing a 50th percentile female in size, was built by modifying/downsizing a 50th percentile male BioRID II dummy. The dynamic response of the BioRID P50F prototype was evaluated with regard to rear impact sled tests comprising female volunteers close to the 50th percentile female size (Carlsson et al., 2021).

### Construction of the BioRID P50F

The BioRID P50F prototype was assembled using modified parts originating from a BioRID II. Target dimensions and masses of the BioRID P50F’s body segments were mainly based on the EvaRID LS-Dyna Model, release version 1.0, by Humanetics (Carlsson et al., 2014). The EvaRID V1.0 model was based on the anthropometric measures of the 50th percentile female from the University of Michigan Transport Research Institute (UMTRI) study (stature 161.8 cm, mass 62.3 kg; Schneider et al., 1983; Table 1).

**TABLE 1:** The length and mass of each body segment of the BioRID II, BioRID P50F, EvaRID model, and the 50th percentile female.

**Dummy segment**	**BioRID II dummy**	**BioRID P50F prototype**	**EvaRID model**	**50th percentile female**
**Length (cm)**				

Head^1^	21.59	19.8	20.30	20.30^8^
Neck^2^	12.04	12.0	10.28	10.28^8^
Torso	52.65^3^	43.8^3^	47.94^3^	41.1^9, 10^
Pelvis	25.83	25.8	25.82	25.82^9^
Arm (upper)^4^	26.14	26.1	26.40	26.4^8^
Arm (lower)^5^	24.88	23.4	23.40	23.4^8^
Leg (upper)^6^	40.55	38.9	38.90	38.9^8^
Leg (lower)^7^	49.55	45.7	45.70	45.7^8^

**Mass (kg)**				

Head	4.44	3.32	3.58	3.58
Torso^11^	27.16	22.43	19.58	19.58
Pelvis	11.67	12.03	15.84	15.84
Arm (upper) × 2	2.02	1.46	1.40	1.40
Arm (lower) × 2	2.26	1.25	1.16	1.15
Leg (upper) × 2	6.86	5.72	5.67	5.68
Leg (lower) × 2	5.80	3.83	3.43	3.43
**Total**	**77.15**	**62.30**	**62.30**	**62.30**

*Additional details can be found in Carlsson et al. (2014). ^1^Top of head to chin. ^2^C0/C1 joint to C7/T1 joint. ^3^C7/T1 joint to mid-point of hip joints. ^4^Shoulder joint to elbow joint. ^5^Elbow joint to wrist joint. ^6^Hip joint to knee joint. ^7^Knee joint to bottom of heel along tibia. ^8^Diffrient et al. (1974). ^9^Young et al. (1983). ^10^“Cervicale landmark” (superior tip of the spine of the 7th cervical vertebra) to “iliocristale landmark” (the highest point on the crest of each ilia in the midaxillary line). ^11^Including neck/spine.*

**FIGURE 1:**
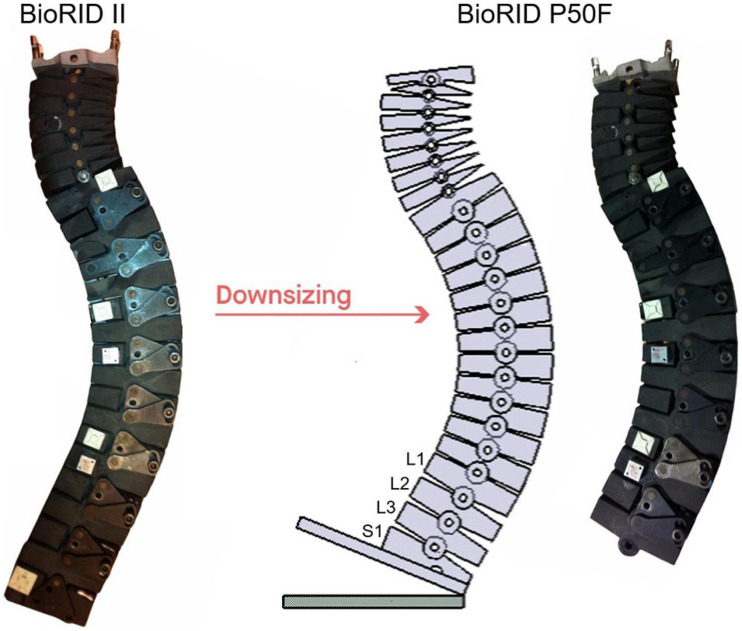
The spine of the BioRID P50F prototype was made by removing two lumbar vertebrae (L4 and L5) from the BioRID II spine and the height of the sacral vertebra (S1) was reduced by 20 mm.

The BioRID’s torso was modified and adjusted to match the overall dimensions and masses of the EvaRID LS-Dyna Model. Two lumbar vertebrae (L4 and L5) were removed from the spine and the height of the sacral vertebra (S1) was reduced by 20 mm (Figure 1). Consequently, the full range of lumbar angular motion was reduced. With these changes, the seated height of the BioRID P50F matched that of the EvaRID model. The construction of the BioRID P50F’s spine, however, deviated from that of the EvaRID model, which has a complete, scaled-down BioRID II spine.

**FIGURE 2:**
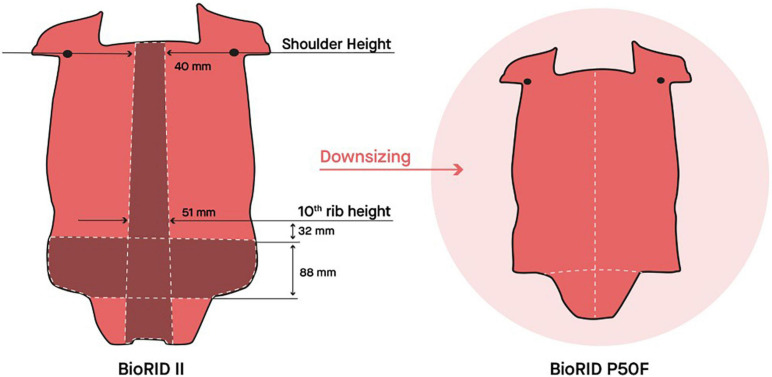
The torso jacket of the BioRID P50F prototype was made by removing the dark segments from the torso jacket of the BioRID II. Illustration courtesy of A. Hedenström.

Two segments were removed from the torso jacket (Figure 2), one mid-sagittal segment to reduce the width of the dummy torso and one horizontal segment in the lower region to reduce the height of the torso. The size of the removed mid-sagittal segment was selected to achieve the same width as the EvaRID model. The shoulder joint and the 10th rib levels were used as landmarks to determine the width of the mid-sagittal segment. At the shoulder joint level, the width of the removed segment was 40 mm, and correspondingly, at the 10th rib level it was 51 mm wide (Figure 2). The size of the removed horizontal segment (88 mm) was selected for the torso jacket to fit the length of the spine. Two-component silicon (Wacker M4601 mixed with a thixotropic stabilizer 43) was used to reassemble the jacket. These modifications resulted in a lateral distance of 305 mm between the shoulder joints. The pins that connect the spine with the jacket were shortened to match the new jacket width.

The stiffness and damping properties of the neck and spine of the EvaRID model were scaled to 70% of the original values in the BioRID II model (Carlsson et al., 2014). This was the starting point for reducing the spine stiffness of the BioRID P50F, however, for practical reasons these reductions did not reach exactly 70%. The polyurethane bumpers, that provide the greatest resistance to flexion and extension of the spine, were decreased: from 15 mm × 10 mm × 10 mm (width × breadth × height) to 10 mm × 10 mm × 10 mm for the anterior and posterior bumpers between C1 and T1; from 25 mm × 15 mm × 2 mm to 20 mm × 15 mm × 2 mm for the posterior bumpers between T1 and T2; from 25 mm × 15 mm × 3 mm to 12.5 mm × 10 mm × 3 mm for the posterior bumpers between T2 and T9; and from 25 mm × 15 mm × 3 mm to 15 mm × 15 mm × 3 mm for the posterior bumpers between T9 and L1. Finally, the springs that control the stiffness of the neck muscle substitute wires (anterior: Stece Die spring No. 51780; L0 = 140.0 mm, C = 9.8 N/mm, posterior: Stece Die Spring No. 51620; L0 = 140.0 mm, C = 16.8 N/mm), were replaced by softer units (anterior: Stece Die spring No. 51820; L0 = 139.7 mm, C = 8.4 N/mm, posterior: Stece Die Spring No. 51780; L0 = 127.0 mm, C = 9.8 N/mm). The design of the spring cartridges and the length of the wires were modified to match the length of the new springs. The wire pretension was in total 14 mm, equivalent to that used with the BioRID II. No modifications were made to the pelvis.

The length of the BioRID II upper arm is 261 mm, measured between the shoulder and the elbow joints. The corresponding length of the EvaRID model is 264 mm (Carlsson et al., 2014, based on Diffrient et al., 1974), thus the length of the upper arms remained unchanged in the BioRID P50F. The lower arms, measured between the elbow and wrist joints, were shortened from 249 to 234 mm, based on Carlsson et al. (2014). Steel skeleton parts were machined, and interior portions of the flesh were removed to reduce mass. The parts reproducing the wrists and hands were removed (Supplementary Appendix Figure A1.1).

**FIGURE 3:**
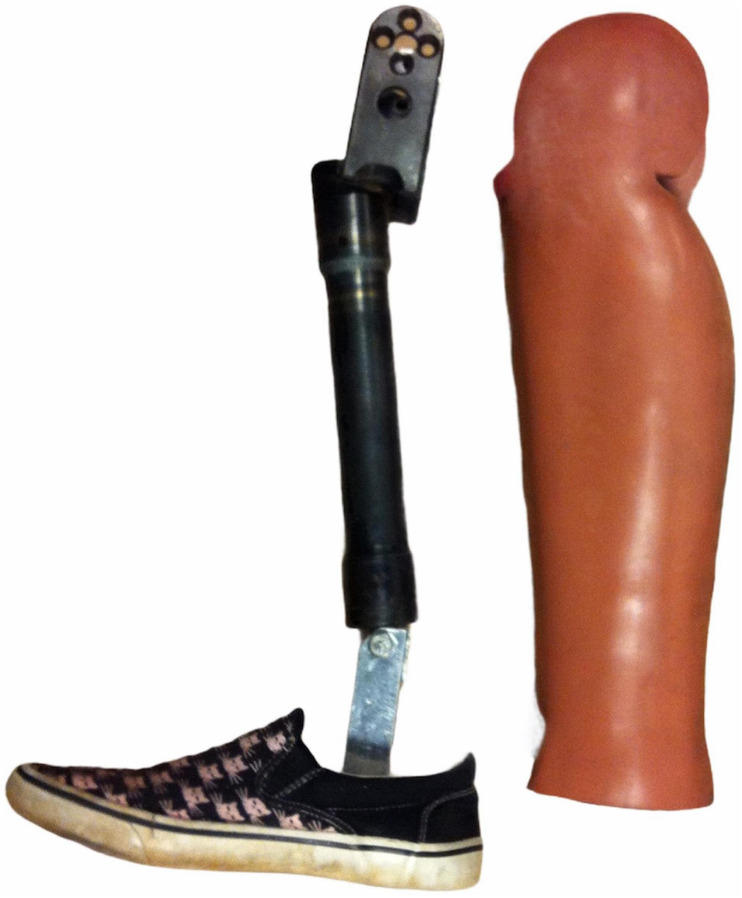
The lower leg of the BioRID P50F prototype.

The original load cell imitations in the upper legs were replaced by 18 mm shorter aluminum cylinders, resulting in an upper leg length of 389 mm, measured between the hip and knee joints, in accordance with the EvaRID model dimensions (Carlsson et al., 2014). The length of the lower legs was reduced from 409 to 376 mm, measured from the knee joint to the ankle joint along the tibia, based on the EvaRID model dimensions (Carlsson et al., 2014). The polymer flesh parts that wrap around the metal parts of the upper and lower legs were cut to match the reduced lengths, and portions of the interior flesh were removed to reduce mass. The BioRID II ankles were replaced by a simplified and lighter design, consisting of an aluminum square profile (25 mm × 25 mm × 2.5 mm) to match the target mass (Figure 3).

**FIGURE 4:**
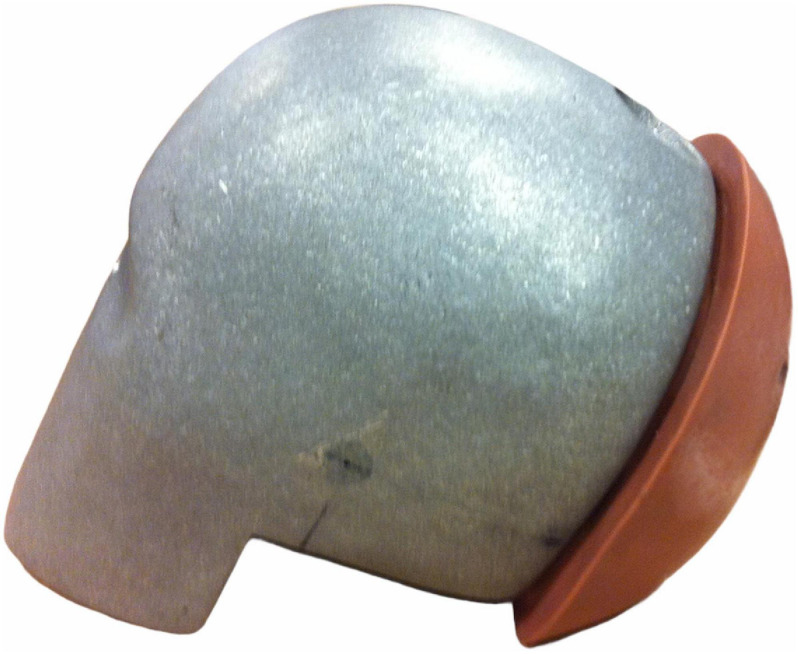
The head of the BioRID P50F prototype.

The BioRID P50F head consisted of a BioRID II head unit with the anterior flesh removed (Figure 4) to match the target mass (Table 1). Body segment dimensions and masses for the BioRID P50F and the BioRID II are listed in Table 1.

### Test With the BioRID P50F Prototype in a Laboratory Seat

**FIGURE 5:**
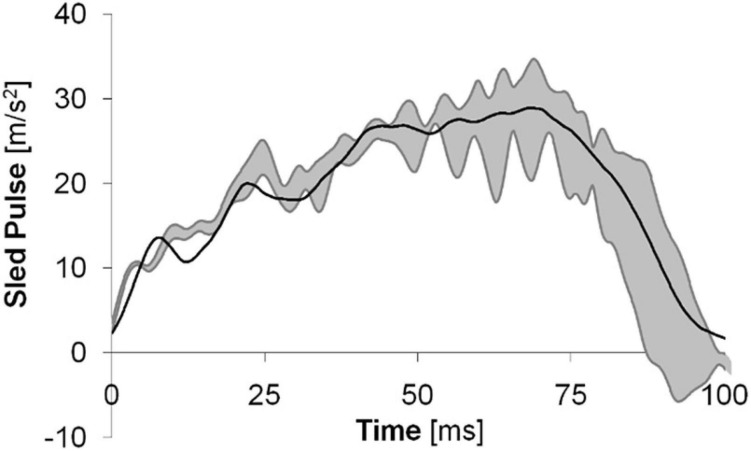
The sled pulse for the tests comprising 50th percentile female volunteers (gray corridor) and the BioRID P50F prototype (solid black line).

In order to evaluate the performance of the BioRID P50F, one test was performed with the prototype dummy in equivalent test conditions as previous tests comprising female volunteers (Carlsson et al., 2021). Eight female volunteers participated in the test series. Their age ranged between 22 and 29 years at an average of 26 years; their stature ranged between 161 and 166 cm at an average of 163 cm; and their mass ranged between 55 and 67 kg at an average of 60 kg. In comparison to the BioRID P50F, the female volunteers were on average 1% taller and 4% lighter. Results from a subset of six volunteer tests at ∼15 cm initial head-to-HR distance, provided in Carlsson et al. (2021), were used as a reference. Carlsson et al. (2021) placed the remaining two tests in a separate category as those volunteers did not contact with the HR. The dummy was seated on a sled, a Hyper-G hydro pneumatic catapult type sled, that was accelerated forward. Figure 5 shows the sled pulse for volunteer tests and the BioRID P50F test.

The BioRID P50F prototype was equipped with three single-axis accelerometers in the head (Endevco 7264C-2k) and a triaxial accelerometer (Meas-spec 1203-500) on the 1st thoracic vertebra (T1). A single-axis accelerometer (ICS/Disynet ICS 3022-200) was attached to the sled base. The coordinate systems were defined according to SAE J211 (orthogonal right-handed). The centers of the accelerometers’ coordinate systems were fixed on the respective accelerometer positions. The head and T1 accelerometers were mounted with initial axes coinciding with the SAE J211 standards.

**FIGURE 6:**
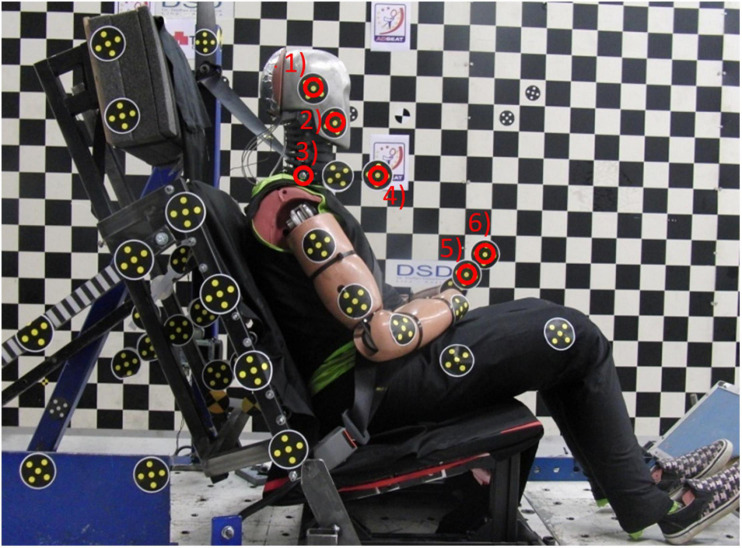
The BioRID P50F prototype in the laboratory seat.

The same laboratory seat was used in this study as in the previous test series with female volunteers (Carlsson et al., 2021). The seatback consisted of four stiff panels covered with 20 mm foam. The panels were independently mounted to a rigid seatback frame by coil springs to allow easy implementation into a computational model. The seatback frame was adjusted to 24.1° from the vertical plane. The HR consisted of a plywood panel covered by firm padding (polyethylene 220-E) and was supported by a stiff steel frame mounted to the seatback. The initial head-to-HR distance was adjusted to 15 cm by adjusting the thickness of the padding (Figure 6). The rigid seat base was angled 16.9° from the horizontal plane. A plate was mounted on the sled to resemble a passenger floor pan surface of a car. The seatback, HR and seat base were covered with double layers of knitted lycra fabric. The pelvic part of the dummy was positioned in accordance with the European New Car Assessment Programme (Euro NCAP) test procedure [European New Car Assessment Programme (EuroNCAP), 2010]. The torso leaned against the seatback, and the T1 as well as the head were aligned with the horizontal plane. The lower arms were positioned on the upper legs (Figure 6).

Film targets were secured on the BioRID P50F and on the seat prior to the tests (Figure 6). Linear displacements of the head and T1 were derived from targets (1) and (3), respectively. Angular displacement of the head and T1 were derived from targets (1) and (2), as well as (3) and (4), respectively. The displacement data were set to zero at the time of impact (T = 0) and were expressed in a sled fixed coordinate system. For practical reasons, the volunteer tests had different target positions to derive the corresponding head and T1 displacements (Carlsson et al., 2021).

The tests were monitored by two high-speed digital video cameras (Kodak RO, 512 × 384 pixels); one providing an overview and one providing a detailed view from the side. The cameras were mounted on a stiff rack attached to the sled, approximately 1.5 m from the volunteers. The frame rate was 1,000 s^–1^ for both cameras. Film targets were digitized using Tema 3.5 software. None of the displacement data were filtered. The data acquisition unit Kayser-Trede MiniDau registered the sensor data at a sampling rate of 20 kHz and the data were filtered in accordance with SAE J211.

The dynamic response of the BioRID P50F prototype was compared to response corridors (the average ± one standard deviation) from the six tests with female volunteers used as reference (Carlsson et al., 2021). The head-to-HR contact time was documented. Additionally, the Neck Injury Criterion (NIC) values (Boström et al., 2000) were calculated.

## Results

**FIGURE 7:**
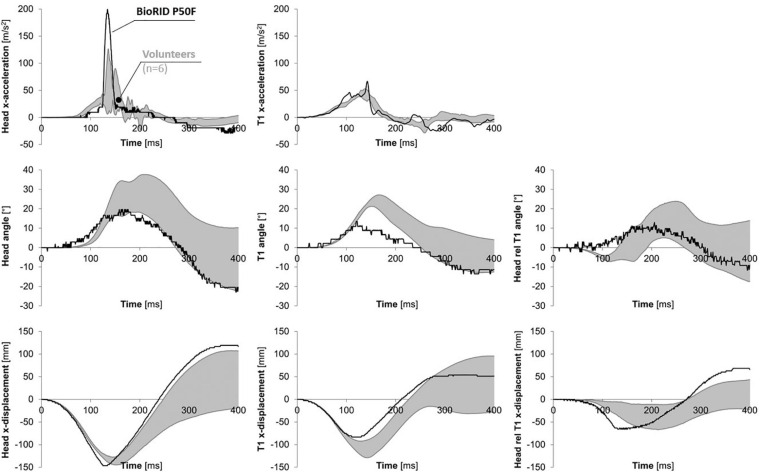
X-acceleration of the head and T1 (NB: rotating coordinate systems); angular displacement of the head, T1 and the head relative to T1; and x-displacement relative to the sled for the head, T1 and the head relative to T1, for the 50th percentile female volunteers (gray corridor) and the BioRID P50F prototype (solid black line). The response corridors were calculated ± 1SD of the average response.

The response of the BioRID P50F prototype dummy in comparison to the volunteer corridors is presented in Figure 7 and Table 2.

**TABLE 2:** Summary of results obtained in the sled test with the BioRID P50F prototype compared with those of female volunteers.

**Variable**	**Volunteers**				**BioRID P50F**	
	**Peak**		**Time**		**Peak**	**Time**
	**Average (SD)**	**Range**	**Average (SD)**	**Range**		
**X-acceleration**	[m/s^2^]	[m/s^2^]	[ms]	[ms]	[m/s^2^]	[ms]
Head	106 (40)	66→173	147 (8)	135→158	199	134
T1	47 (6)	38→54	135 (13)	113→153	67	142
**NIC**	[m^2^/s^2^]	[m^2^/s^2^]	[ms]	[ms]	[m^2^/s^2^]	[ms]
	5.0 (2.1)	3.0→7.8	123 (23)	79→141	8.5	106
**Ang. displacement**	(°)	(°)	[ms]	[ms]	(°)	[ms]
Head	28 (9)	16→38	202 (13)	185→216	20	167
T1	24 (3)	21→30	159 (8)	151→174	13	132
Head relative to T1^2^	−7 (2)	−9→−4	126 (27)	99→164	–	–
Head relative to T1^3^	15 (9)	5→26	235 (15)	212→258	12	200
**X-displacement**^1^	[mm]	[mm]	[ms]	[ms]	[mm]	[ms]
Head	−138 (9)	−147→−125	149 (7)	141→156	−147	130
T1	−103 (9)	−145→−94	136 (7)	129→145	−86	123
Head relative to T1	−52 (12)	−65→13	187 (36)	147→233	−63	132
**Head restraint**	[mm]	[mm]	[ms]	[ms]	[mm]	[ms]
Distance^4^	144 (6)	135→153	–	–	150	–
Contact	–	–	129 (8)	118→139	–	120

*^1^Relative to the sled. ^2^First peak. ^3^Second peak. ^4^At T = 0 ms (based on film analysis).*

The BioRID P50F’s head remained stationary for a longer time which delayed the head x-acceleration onset compared to the volunteers. This led to an earlier rise in head x-displacement and a greater and somewhat earlier peak head x-acceleration for the BioRID P50F prototype compared to the volunteers. The T1 x-acceleration was similar for the volunteers and the BioRID P50F prototype during the first ∼85 ms. As the upper torso of the BioRID P50F prototype was pushed forward by the seatback, the T1 x-acceleration began to increase, peaking (142 ms) as the head reached the HR. The NIC value was on average 70% greater and occurred 13% earlier for the BioRID P50F prototype (8.5 m^2^/s^2^ at 106 ms), compared to the female volunteers (5.0 m^2^/s^2^ at 123 ms) (Table 2).

The head rearward angular displacement of the BioRID P50F prototype was close to the corridor of the female volunteers, however, the onset began somewhat early. The peak T1 rearward angular displacement was lower and earlier for the BioRID P50F prototype compared to the female volunteers. The volunteers exhibited a small flexion of the head relative to the T1 angular displacement during the first ∼160 ms, since the rearward angular displacement of T1 began earlier than that of the head. This small flexion was not found in the BioRID P50F prototype due to the early onset of the head angular displacement. As the volunteers’ heads began to rotate rearward, the flexion of the head relative to T1 changed into extension. The corresponding extension angle for the BioRID P50F prototype was within the corridor of the female volunteers.

The BioRID P50F rearward head x-displacement relative to the sled was similar to that of the volunteers. However, the peak occurred somewhat earlier for the BioRID P50F prototype due to the earlier head-to-HR contact, which in turn can be explained by the earlier head x-displacement. Compared to the volunteers, the rearward x-displacement of the T1 was slightly less for the BioRID P50F prototype.

## Discussion

The aim of this study was to present the design of the prototype rear impact crash test dummy, representing a 50th percentile female. The BioRID P50F prototype was assembled using BioRID II dummy parts, modified/downsized to match the anthropometric dimensions and mass distribution of the 50th percentile female (Carlsson et al., 2014; Table 1). BioRID P50F is thus based on the same design principle as the BioRID II. Previous studies have shown that the BioRID II is a highly repeatable and reproducible dummy design (Eriksson and Zellmer, 2007). Therefore, for the purpose of the present study, it was considered sufficient for performing a single test, exclusively. The head/neck response of the BioRID P50F prototype resembled the female volunteer response corridors (Figure 7).

The BioRID P50F included a BioRID II spine where two lumbar vertebrae (L4 and L5) were removed and the height of the S1 was reduced. Hence, the full range of lumbar angular motion was decreased. However, this had minor influence since the lumbar angular motion was restricted in the rear impact load case due to the support from the seatback. Furthermore, the thoracic and lumbar pin joint stiffnesses as well as the depth of the dummy torso of the BioRID II was kept in the BioRID P50F prototype. Therefore, it is most likely that the torso and thoraco-lumbar spine segments were stiffer than in an average female. Consequently, the rearward angular and x-displacements of the T1 were less for the BioRID P50F prototype in comparison to the female volunteers (Figure 7 and Table 2). Furthermore, the NIC value was 70% greater in the BioRID P50F compared to the volunteers, which reflects the head and T1 x-accelerations (Figure 6) at the NIC peak at 106 ms (Table 2). In comparison to the volunteer x-accelerations at 106 ms, the amplitude of the head was 59% less, while the amplitude of the T1 was 57% greater, both contributing to a greater NIC value. The biofidelity of the BioRID P50F prototype thus have some limitations.

**FIGURE 8:**
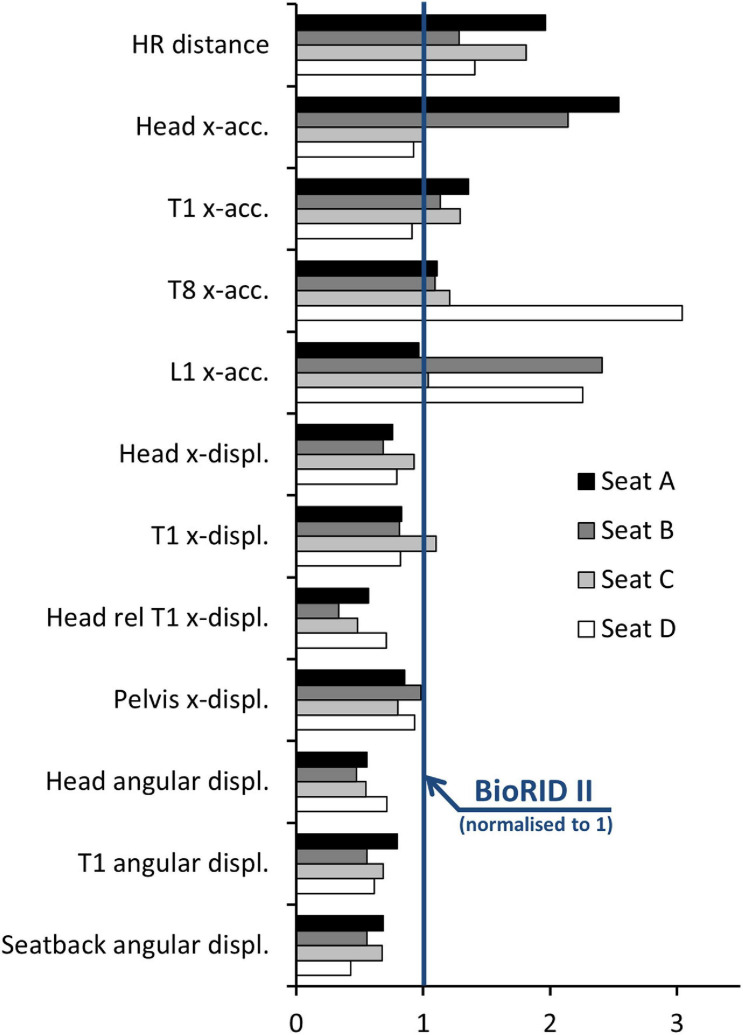
Results from tests with the BioRID P50F prototype in four different standard seats (A–D) normalized with respect to the corresponding BioRID II Euro NCAP results (solid blue line); picture from Carlsson (2012).

Despite these limitations, we realized that the BioRID P50F can be used to determine whether the male BioRID II also sufficiently represent the average female, in the assessment of the dynamic seat and HR response. This was investigated in a study by Schmitt et al. (2012), demonstrating the difference in seat interaction between the average male and female dummy sizes. A rear impact test series was performed in four different standard vehicle seats (A–D). Seats A, B and D were equipped with different types of whiplash protection systems, while Seat C was made in a basic seat design. According to Euro NCAP, Seats A, B and D were rated good, and Seat C performed marginally. Results comparing the BioRID P50F response with previously reported results for the BioRID II were presented by Carlsson (2012) (Figure 8) as well as by Schmitt et al. (2012) (Supplementary Appendix Figure A1.2).

Different trends were found for different seat models when comparing the female and male dummy responses (Figure 8 and Supplementary Appendix Figure A1.2). The results indicate that there is no simple way to “reinterpret” or “scale” BioRID II data to address the female dynamic response. A fully validated 50th percentile female rear impact dummy would not only be an important tool for the design and evaluation of future protective systems, but also a tool useful in the process of further development and evaluation of injury criteria. An average female rear impact dummy could, together with the existing average male dummy, be used to complement the studies of Kullgren et al. (2003) and Linder et al. (2004) to find neck injury threshold values for female and male dummies separately. As a first estimate, Linder et al. (2013) suggested reducing the NIC threshold value from 15 to 12 m^2^/s^2^ for the average sized female. Furthermore, it was suggested to reduce the intercept values of the N_km_ from 47.5 Nm to 29 Nm for extension moment, from 88.1 to 53 Nm for flexion moment and from 845 N to 507 N for shear force, for the average sized female.

In the present study, the head-to-HR distance was adjusted to 15 cm, in accordance with the tests with female volunteers (Carlsson et al., 2021). It is important to note that this HR adjustment procedure deviates from the typical situation in a standard passenger vehicle seat. The study of Schmitt et al. (2012) compared the BioRID P50F to the BioRID II in four different standard seats. They reported a 28–96% greater head-to-HR distance for the BioRID P50F compared to the BioRID II (Figure 8). However, based on volunteer tests in seats without horizontal head-to-HR distance adjustment it has been reported that the head-to-HR distance is shorter for 50th percentile females than 50th percentile males (Welcher and Szabo, 2001; Linder et al., 2008; Carlsson et al., 2010; Carlsson et al., 2011; Carlsson et al., 2017). The greater head-to-HR distance for BioRID P50F may be a result of its thoracic spinal curvature, which is taken directly from the male BioRID II dummy. Sato et al. (2016) observed that the female thoracic spine curvature is far less kyphotic compared to the male. This suggests that the BioRID P50F T1 vertebra position is too far forward compared to an average female.

To conclude, the overall response of the BioRID P50F prototype dummy resembled the female volunteer response corridors in low severity rear impacts. However, further refinements and additional validations would be needed in order to bring it to the same level of biofidelity as the BioRID II. This would include improved surface geometry and local mass distribution, as well as a modified spine, more representative of female properties with regards to the number of vertebrae, stiffness, curvature and range of motion. The targeted reduction to 70% of the spinal stiffness, in the present study, requires further tuning to match the outcome of the volunteer tests. Furthermore, sensor equipment corresponding to that of the BioRID II can be used in the present BioRID P50F. Additional validations could include alternative volunteer data sets, as well as Post Mortem Human Subject (PMHS) testing at higher impact severity. In future it would be of high value to have 50th percentile rear impact dummies of both the female and the male sizes. Ideally, these two dummy versions should be based on the same design principles and have the same level of biofidelity, which could be ensured by comparison to volunteer response data, using biofidelity ratings such as correlation and Analysis (CORA) or similar. The BioRID II represents the 50th percentile male which limits the assessment and development of whiplash protection systems with regard to female occupants. It is therefore important that future whiplash protection systems are developed and evaluated taking female properties into account. Thus, the need to develop a new fully validated rear impact 50th percentile female dummy, suitable for use in parallel with the current male dummy, is apparent.

## Data Availability Statement

The raw data supporting the conclusions of this article will be made available by the authors, without undue reservation.

## Author Contributions

AC: preparation, execution, documentation, and analysis of the test series. JD: preparation of the test series, advice based on earlier experience in experimental whiplash injury research including crash test dummy development, and internal review of the manuscript. AL: EU project coordinator, planning of the test series, advice based on earlier experience in experimental whiplash injury research, and internal review of the manuscript. MS: principal investigator, WP leader in the two involved EU projects, initiated the work in the present study, and contributed with advice based on earlier experience in experimental whiplash injury research including crash test dummy development. All authors contributed to the article and approved the submitted version.

## Conflict of Interest

The authors declare that the research was conducted in the absence of any commercial or financial relationships that could be construed as a potential conflict of interest.
