# Supplementary material for: Design and Evaluation of the Initial 50th Percentile Female Prototype Rear Impact Dummy, BioRID P50F – Indications for the Need of an Additional Dummy Size
Source: Front Bioeng Biotechnol. 2021 Jul 16;9:687058. doi: 10.3389/fbioe.2021.687058 (PMC8322785; doi:10.3389/fbioe.2021.687058)
Supplement: Supplementary file 1 [file Data_Sheet_1.PDF]

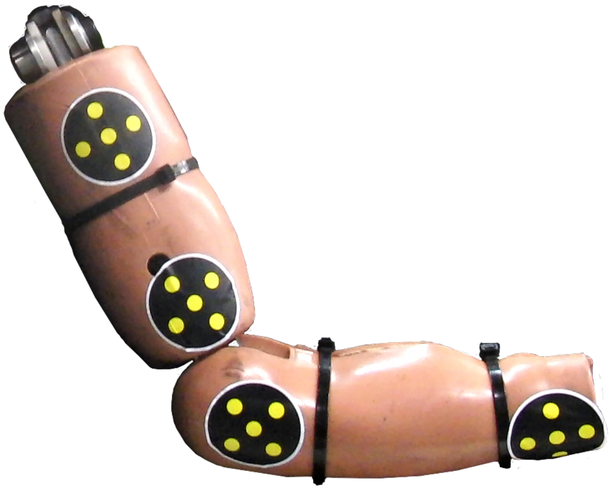

Figure A1.1. *The arm of the BioRID P50F prototype.*

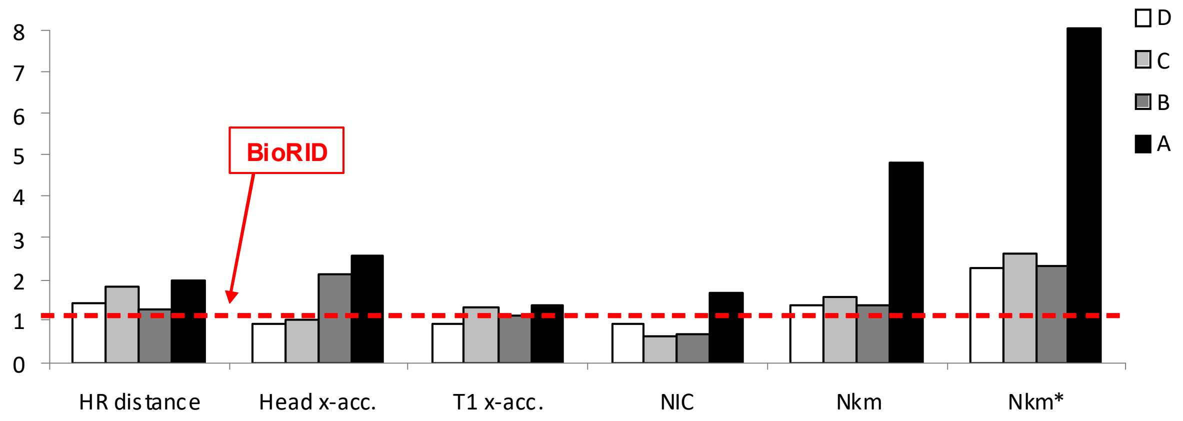

Figure A1.2. *Test results normalised with respect to the corresponding Euro NCAP results. Picture from Schmitt et al. (2012).*
